# Exposure to Urinary and Dust Parabens: Compound-Specific Risks for Pediatric Respiratory Allergic Phenotypes

**DOI:** 10.3390/toxics14040281

**Published:** 2026-03-26

**Authors:** Yangyang Zhu, Shuang Du, Zhiqi Lin, Qingshuang Li, Hao Tang, Zhiping Niu, Dan Norbäck, Tippawan Prapamontol, Chanjuan Sun, Jiufeng Li, Zhuohui Zhao

**Affiliations:** 1 Department of Environmental Health, School of Public Health, Fudan University, Shanghai 200032, China; 23211020093@m.fudan.edu.cn (Y.Z.); 22111020050@m.fudan.edu.cn (S.D.); 24211020143@m.fudan.edu.cn (Z.L.); 22111020056@m.fudan.edu.cn (H.T.); 23111020059@m.fudan.edu.cn (Z.N.); 2 School of Environment and Architecture, University of Shanghai for Science and Technology, Shanghai 200093, China; 232291905@st.usst.edu.cn (Q.L.); sunchanjuan@usst.edu.cn (C.S.); 3 Department of Occupational and Environmental Medicine, Uppsala University Hospital, 751 85 Uppsala, Sweden; dan.norback@medsci.uu.se; 4 Research Institute for Health Sciences, Chiang Mai University, Chiang Mai 50200, Thailand; tprapamontol@gmail.com; 5 Department of Environmental Health, School of Public Health, NHC Key Laboratory of Health Technology Assessment, Key Laboratory of Public Health Safety of the Ministry of Education, Fudan University, Shanghai 200032, China

**Keywords:** parabens, allergic rhinitis, asthma, comorbidity, dust, indoor environment

## Abstract

Parabens, a prevalent class of endocrine-disrupting chemicals (EDCs), are ubiquitous in consumer products; however, their role in linking pediatric allergic phenotypes remains poorly understood. This case-control study analyzed paraben levels in urine and indoor dust as proxies for internal and external exposures and investigated their associations with allergic rhinitis only (AR Only), asthma only (AS Only), and comorbidities (AR&AS) among children in Shanghai. The concentrations for each of four paraben compounds were quantitatively measured, and multi-pollutant frameworks—including Bayesian Kernel Machine Regression (BKMR) and Weighted Quantile Sum (WQS) regression—were employed to characterize the mixture exposure and risk. Propylparaben (PrP) was detectable in 100% of urine samples and over 90% of dust samples, and the concentrations ranked the highest out of the four compounds in both samples. Benzylparaben (BzP) was detected in >70% of urine samples and over 50% of dust samples at relatively lower levels. Urinary PrP exhibited significantly positive associations with all phenotypes (OR in 2.18–2.92) and BzP with the AR&AS Comorbidity (OR = 3.55, 95% CI: 1.32–9.55). Dust-borne PrP was associated with AR Only (OR = 2.26, 95% CI: 1.16–4.43), indicating a potential “Portal of Entry” effect via direct nasal deposition. According to BKMR and WQS analyses, urinary PrP and BzP emerged as two primary risk drivers. Using interaction analysis, an additive synergistic effect was observed between urinary PrP and BzP with parental history of allergy, suggesting heightened vulnerability to paraben exposure in genetically predisposed subgroups. In conclusion, children with respiratory allergies were associated with higher exposure to PrP and BzP and exhibited higher susceptibility in those with a parental history of allergy.

## 1. Introduction

In the modern chemical landscape, humans are exposed to a complex mixture of anthropogenic compounds, many of which function as endocrine-disrupting chemicals (EDCs) capable of perturbing hormonal homeostasis [1,2,3]. Among these, parabens represent a pervasive source of continuous exposure. Valued for their broad-spectrum antimicrobial efficacy and stability, these compounds are extensively employed in personal care products (PCPs), pharmaceuticals, and processed foods [4,5].

Exposure to parabens can occur through multiple pathways, including dermal absorption, dietary ingestion, and the inhalation or ingestion of indoor dust. Urinary samples can be a reservoir for the total internal exposure, which sums up the multi-exposure pathway [6,7]. Global biomonitoring data have demonstrated widespread human exposure to parabens, with their metabolites consistently detected in urine samples across diverse populations in the United States, China, and Europe [8,9,10,11]. There is a rapid biological clearance of parabens—typically characterized by half-lives under 24 h. However, it is effectively countered by the near-continuous nature of consumer product usage, resulting in a ‘pseudo-persistent’ internal dose that poses chronic health risks [12,13].

From a toxicological perspective, the potential for parabens to drive adverse health outcomes is biologically plausible but heavily structure-dependent. The primary concern stems from their ability to mimic natural estrogens and bind to estrogen receptors (ERs) [14,15,16]. Crucially, this endocrine potency increases with the length and bulkiness of the ester side chain [17,18]. While the short-chain methylparaben (MeP) is abundant, its activity is relatively weak. In contrast, the congeners prioritized—ethylparaben (EtP), propylparaben (PrP), butylparaben (BuP), and benzylparaben (BzP)—exhibit significantly greater lipophilicity and stronger receptor binding affinity [19,20]. BzP, in particular, possesses an aryl group that confers estrogenic potency magnitudes higher than short-chain alkyl esters [14,21], yet it remains frequently overlooked in standard screening panels. Beyond hormonal disruption, emerging evidence points to an “endocrine-immune interface,” where these potent EDCs may modulate immune trajectories by skewing the Th1/Th2 cytokine balance towards a Th2 profile, theoretically lowering the threshold for allergic sensitization [1,22,23,24]. In vitro assays have further demonstrated that parabens, particularly BzP and PrP, can activate human eosinophils, enhance histamine release from mast cells, and potentiate oxidative stress, thereby theoretically lowering the threshold for allergic sensitization [25,26].

Despite this strong biological rationale, epidemiological evidence linking paraben exposure to allergic diseases remains inconsistent. Allergic rhinitis (AR), Asthma (AS), and comorbidity (AR&AS) are common respiratory allergic diseases in children, which show an increasing prevalence, particularly in younger generations, amidst ongoing urbanization. While some studies reported positive associations between urinary PrP or BuP and asthma [10,24], others found null or even inverse associations [23,27,28]. Behind the inconsistency, there may be several reasons being neglected. Children are exposed to complex mixtures, not single compounds; therefore, traditional single-pollutant models failed to account for the collinearity and interactive effects of co-occurring congeners. Exposure assessment relying solely on spot urine samples may misclassify long-term exposure, whereas environmental matrices, like settled dust, can supplement and reflect integrated exposure over longer durations. Regarding health outcomes, the “United Airway Disease” (UAD) hypothesis unifies the upper and lower respiratory tracts and suggests that up to 80% of asthmatics have co-occurring AR [29,30,31,32,33]. By treating asthma and AR as isolated entities only, previous studies might have diluted observable environmental effects and masked risks specific to the most severely affected subgroups [30,34,35].

Thus, we conducted a comprehensive case-control study in children in Shanghai, China, by integrating measurements of four toxicologically relevant parabens (EtP, PrP, BuP, and BzP) across two sample types: urine (internal) and settled dust (external). Utilizing advanced chemicals analysis method and mixture statistics—specifically Bayesian Kernel Machine Regression (BKMR) and Weighted Quantile Sum (WQS) regression—our objectives were to: (1) characterize the co-exposure profiles of these parabens; (2) elucidate their individual and joint associations with three respiratory Allergic Phenotypes (AR Only, Asthma Only, and AR&AS Comorbidity); and (3) explore effect modifications by a parental history of allergy. Children are particularly vulnerable to EDCs exposure, not only due to developing physiological systems that are less efficient at detoxification but also due to distinct behavioral patterns, such as frequent hand-to-mouth activity, which amplify their contact with environmental media [36,37]. This study aims to provide phenotype-specific evidence to inform paraben risk assessment to improve children’s environmental health.

## 2. Materials and Methods

### 2.1. Study Subjects

This case–control study was conducted between 2019 and 2023 and enrolled children who had lived in Shanghai for at least 12 months prior to recruitment. Cases were diagnosed by clinical physicians following the Consensus on *Diagnosis and Management of Allergic Diseases in Children* [38] and stratified into three phenotypes based on clinical presentation: (1) Allergic Rhinitis Only (AR Only), (2) Asthma Only (AS Only), and (3) Allergic rhinitis-Asthma Comorbidity (AR&AS Comorbidity). The control group, matched by gender proportion at similar ages (±12 months), comprised children with no clinical history of allergies and no respiratory diseases in the preceding 12 months. Children with known congenital disorders or other significant chronic illnesses were excluded from both groups. The survey was approved by the Medical Ethics Committee of Ruijin Hospital (IRB#2019-09-0778), and informed consent has been obtained from parents/legal guardians of subjects.

### 2.2. Data and Sample Collection

Upon enrollment, guardians completed a structured questionnaire adapted from the International Study of Asthma and Allergies in Childhood (ISAAC). Data was collected on demographics, clinical history, history of medication use, socioeconomic status (SES), parental smoking, and indoor environmental characteristics. The study included 366 participants, comprising 246 pediatric cases and 120 healthy controls. The case group consisted of 105 children with AR Only, 66 with AS Only, and 75 with AR&AS Comorbidity.

About 20 mL spot urine samples were collected from each participant during home visits. To minimize background contamination, samples were collected in pre-screened, paraben-free polypropylene tubes. Samples were immediately placed in portable coolers (4 °C), transported to the laboratory within 4 h, and stored at −80 °C until analysis.

Concurrent with biological sampling, indoor settled dust was collected to assess external exposure. Prior to sampling, parents were instructed not to clean the child’s bedroom for at least three consecutive days. Dust samples were collected using a customized vacuum cleaner equipped with a nylon filter membrane. To prevent cross-contamination, the sampling head was disinfected with 70% alcohol and air-dried before each use. A composite sample was collected from multiple surfaces (floors, mattresses, curtains, desktops, and sofas) within the bedroom with a standardized active sampling time of 30 min. Filters containing dust were wrapped in aluminum foil, placed in sealed polyethylene bags, and stored at −80 °C. Dust was sieved through a 500 µm mesh to obtain the fine fraction for analysis.

A total of 275 urine samples were collected, including 93 samples from controls and 182 from cases. Additionally, 314 dust samples were collected, of which 101 were from controls and 213 from cases.

### 2.3. Chemical Analysis

The sample preparation and instrumental analysis were conducted using a laboratory-developed analytical method that was internally validated in our laboratory.

#### 2.3.1. Sample Preparation

Total concentrations of parent parabens (free and conjugated) in urine samples were quantified following enzymatic hydrolysis to capture the biologically active diesters and ensure homologue specificity, thereby avoiding the confounding influence of the non-specific and less potent metabolite PHBA.

In detail, a 0.4 mL aliquot of urine was spiked with 100 μL of an isotope-labeled internal standard mixture prior to the addition of the 100 μL β-glucuronidase solution (Helix pomatia, >85,000 units/mL, Sigma-Aldrich, Saint Louis, MO, USA; buffered in 1 M ammonium acetate, pH 5.0). Samples were incubated at 37 °C for 16 h with agitation (50 rpm). Post-hydrolysis analytes were extracted via liquid–liquid extraction (LLE) using 3 mL of ethyl acetate acidified with 0.1% formic acid. The mixture was vortexed (5 min) and centrifuged (4000 *g*, 10 min). This extraction was repeated three times. The combined organic extracts were evaporated to dryness under a gentle nitrogen stream at 25 °C and reconstituted in 300 μL of methanol–water (6:4, *v*/*v*). Urinary creatinine (U-Cre) was determined using a sarcosine oxidase assay (Nanjing Jiancheng Bioengineering Institute) to adjust for dilution.

The sieved dust samples were pretreated before the instrumental analysis. Dust samples were sieved through a mesh with a pore size of 500 µm prior to extraction. An aliquot of 50 ± 0.4 mg of dust was weighed into a glass centrifuge tube and spiked with 100 μL of the internal standard solution. Extraction was performed by adding 3 mL of ethyl acetate, vortexing for 5 min, and centrifuging at 2500 rpm for 10 min. The supernatant was transferred to a new tube. This process was repeated twice, combining the supernatants (total volume ~9 mL). The extract was evaporated to dryness under nitrogen. The residue was reconstituted in 500 μL of 90% acetonitrile. The solution was purified using an Amicon^®^ Ultra-4 centrifugal filter unit (10 kDa MWCO, Millipore, Billerica, MA, USA) by centrifugation at 3000 rpm for 20 min. The filtrate was transferred to a vial for HPLC-MS/MS analysis.

#### 2.3.2. Instrumental Analysis

Target analytes (PrP, BzP, EtP, and BuP) were quantified using a Waters ACQUITY UPLC system coupled to a XEVO TQ-S MICRO triple quadrupole mass spectrometer (Waters Corp., Milford, MA, USA). Separation was achieved on an ACQUITY UPLC BEH C18 column (2.1 × 100 mm, 1.7 µm; 40 °C) using a gradient mobile phase of (A) 2 mM aqueous ammonium acetate (pH 4.6–5.0) and (B) acetonitrile (Table S1). Detection was performed in negative electrospray ionization (ESI-) mode using Multiple Reaction Monitoring (MRM). Source parameters and transition definitions are detailed in Table S2.

#### 2.3.3. Quality Assurance and Quality Control (QA/QC)

To strictly control contamination, all glassware was solvent-rinsed with absolute ethanol prior to use. Each analytical batch included procedural blanks, solvent blanks, and matrix-spiked samples. Field blank samples were also collected to monitor potential contamination during sample transport and handling. Quantification was performed using an isotope dilution calibration method, with calibration curves showing good linearity (R^2^ > 0.99). Method validation for urine was conducted using phosphate-buffered saline (PBS) as a surrogate matrix. Spike-recovery experiments were performed at 5, 20, and 40 ng/mL (*n* = 6), and the recoveries ranged from 85% to 120%, with relative standard deviations (RSDs) below 15%. The limits of detection (LODs) and limits of quantification (LOQs) were defined at signal-to-noise ratios of 3 and 10, respectively. Concentrations below the LOD were imputed as LOD/√2, while concentrations between the LOD and LOQ were assigned as LOQ/√2.

The quality control procedures for dust samples were generally consistent with those used for urine samples. Method validation for dust was conducted using anhydrous sodium sulfate as a surrogate matrix. Spike-recovery experiments were performed at 5, 10, and 40 ng/mL (*n* = 6). Detailed method validation parameters for both urine and dust matrices are provided in Table S3.

Detailed method validation parameters are presented in Table S3.

### 2.4. Statistical Analysis

Statistical analyses were performed using R (version 4.4.0). Urinary concentrations were creatinine-corrected and log_10_-transformed to improve normality.

The associations between paraben exposure and overall pediatric allergic cases, as well as each of the three specific phenotypes, were evaluated using binary logistic regression. For each paraben, participants were categorized into high and low exposure groups using the median concentration as the cutoff point. The low exposure category was defined as the reference. The covariates were determined based on the DAG diagram (Figure S1). Models were adjusted for age, gender, residence (urban/rural), parental education, parental allergy history, air purifier usage frequency, and bedroom cleaning frequency.

To assess mixture effects, BKMR and WQS regression were applied. BKMR (probit link, 30,000 iterations) elucidated non-linear associations and interactions, using Posterior Inclusion Probabilities (PIPs) to pinpoint key contributors. The PIP threshold over 0.5 was considered to be a key driver factor. WQS regression constructed a weighted index of the mixture effect based on 1000 bootstrap samples. It reveals the cumulative health effect of the paraben mixture. Additionally, biological interaction was evaluated with parental history of allergies using the interactionR package in R version 4.5.1. A two-tailed *p* < 0.05 indicated statistical significance.

## 3. Results

### 3.1. Demographics and Household Characteristics

**Figure 1:**
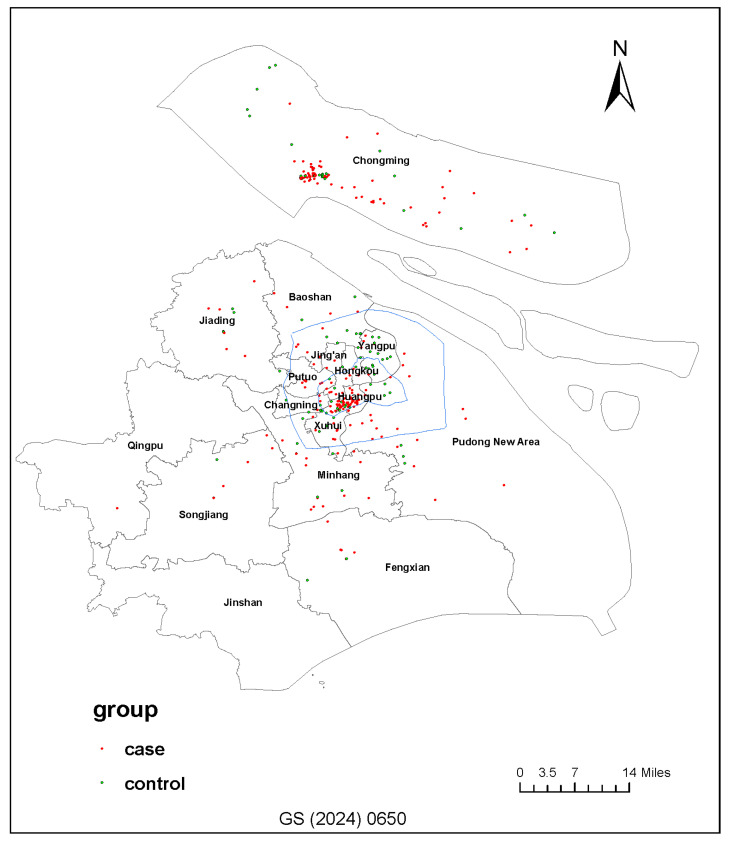
Distribution map of residences of pediatric case–control group in Shanghai (GS). Spatial distribution of residential locations for children in the case (red dots) and control (green dots) groups across the administrative districts of Shanghai, illustrating the coverage of both urban and suburban areas.

The spatial distribution of the study population in Shanghai is shown in Figure 1. The mean age of all participants was approximately 7.1 ± 2.6 years (cases: 7.2 ± 2.6 years; controls: 6.9 ± 2.6 years). Compared with controls, the case group had a significantly higher proportion of boys (67.1% vs. 53.8%, *p* = 0.019), indicating that male children were more frequently represented among cases in this study population (Table 1). In addition, parents in the case group generally had a lower educational level, with a higher proportion classified as undergraduate and below (74.0% vs. 55.0%, *p* < 0.001). Cases also had a higher prevalence of parental history of allergies than controls (60.2% vs. 28.3%, *p* < 0.001), suggesting a possible familial predisposition. A higher proportion of cases were also exposed to parental smoking history compared with controls (44.1% vs. 32.2%, *p* = 0.041). Detailed demographic and household characteristics are presented in Table 1.

### 3.2. Urine Paraben Compounds and Associations with Respiratory Allergic Phenotypes

Among four parabens (PrP, BzP, EtP, and BuP), PrP was ubiquitously detected in all urine samples with a 100% detection rate (DR) and had the highest detection concentration, while BzP and EtP were detected in 79.7% and 66.5% of cases, respectively, higher than those of controls (71.0% and 54.8%) (Table S4). BuP was detected in around 41% of urine samples, similar between cases and controls.

**Figure 2:**
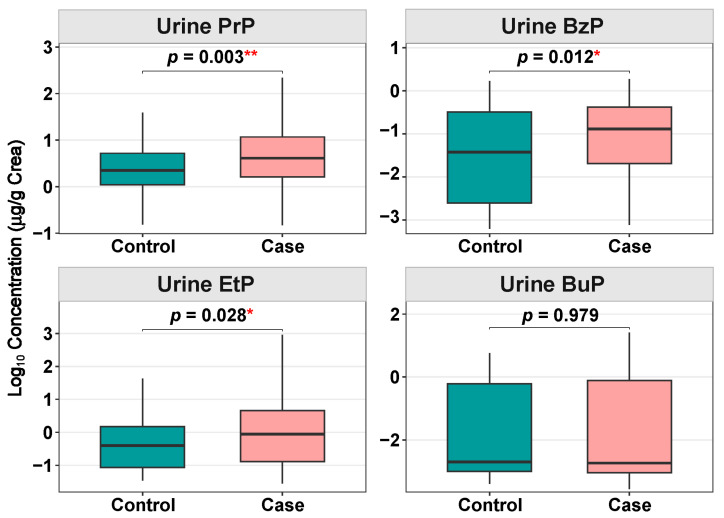
Distribution of four parabens in urine samples. Comparison of creatinine-corrected and log10-transformed urinary concentrations of EtP, PrP, BuP, and BzP between the “All Case” group and Controls. *p*-values are derived from the Mann–Whitney U test. Detection Rate (DR) indicates the percentage of samples exceeding the Limit of Detection (LOD). * *p* < 0.05; ** *p* < 0.01.

**Figure 3:**
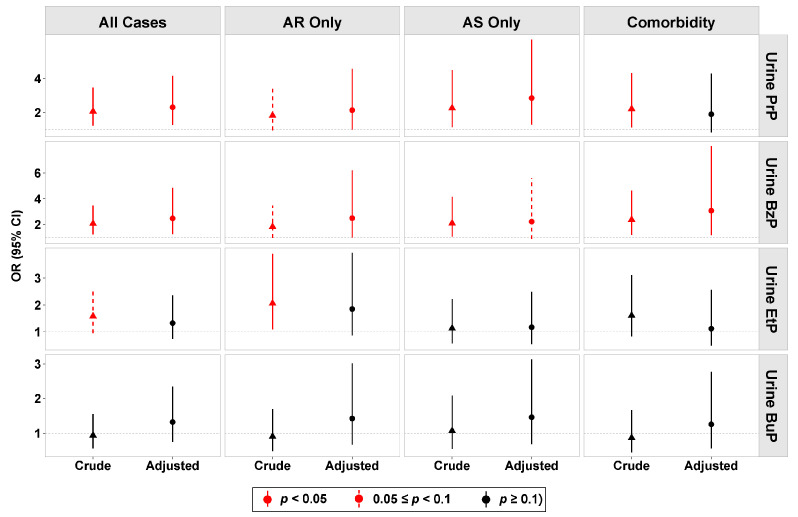
Logistic regression analysis of urinary parabens and respiratory allergy phenotypes. Forest plot displaying adjusted Odds Ratios (ORs) with 95% Confidence Intervals (CIs). High-exposure groups (>P50) are compared to low-exposure groups (<P50, reference) by the median level. Urinary paraben concentrations were log10-transformed and creatinine-corrected. Models are adjusted for age, sex, parental education, parental allergy history, residence, air purifier use, and bedroom cleaning frequency. Red solid line: *p* < 0.05; red dashed line: 0.05 ≤ *p* < 0.1; black solid line: *p* ≥ 0.1.

Compared with controls, creatinine-corrected urine PrP, BzP, and EtP were significantly higher in the case group (Figure 2). According to logistic regression analyses, PrP and BzP demonstrated robust and pervasive associations with almost all phenotypes, both in the crude and adjusted models (Figure 3; Table S5). Conversely, associations for EtP and BuP showed no statistical significance across the three phenotypes.

Urine PrP was significantly associated with “All Cases” by an adjusted OR (aOR) of 2.37 (95% CI: 1.32–4.25, *p* = 0.004), with “AR Only” by an aOR of 2.18 (95% CI: 1.02–4.65, *p* = 0.043), with “AS Only” by an aOR of 2.92 (95% CI: 1.33–6.42, *p* = 0.008), and a numerically positive but not significant association for AR&AS Comorbidity, respectively, after adjusting for potential confounders, including age, gender, residence (urban/rural), parental education, parental allergy history, air purifier usage frequency, and bedroom cleaning frequency.

BzP exhibited a similar strong association with “All Cases” (aOR = 2.61, 95% CI: 1.33–5.13, *p* = 0.005), “AR Only” (aOR = 2.53, 95% CI: 1.02–6.28, *p* = 0.046), and “AR&AS Comorbidity” (aOR = 3.55, 95% CI: 1.32–9.55, *p* = 0.012). Notably, distinct from PrP, the effect of BzP was particularly pronounced in the Comorbidity subgroup, where the aOR reached the highest. It indicated that BzP might correspond to a more complex clinical phenotype involving inflammation in both the upper and lower airways.

### 3.3. Urine Paraben Mixture and Associations with Respiratory Allergic Phenotypes

**Figure 4:**
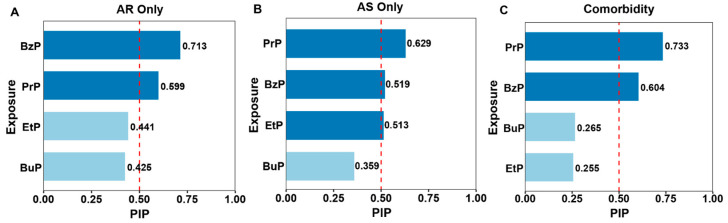
BKMR analysis of urine paraben mixture effects. Bar plots represent the Posterior Inclusion Probability (PIP) for each paraben across disease subgroups: (**A**) AR Only, (**B**) AS Only, and (**C**) AR&AS Comorbidity. The dashed line indicates a PIP threshold of 0.50. Dark blue bars represent the key contributors (PIP ≥ 0.50) to the mixture effect, while light blue bars indicate variables with lower contribution (PIP < 0.50).

In the BKMR analysis, the differential contribution of individual compounds to target health outcomes was highlighted (Figure 4). BzP and PrP consistently exceeded the Posterior Inclusion Probability (PIP) threshold of 0.50 for 3 phenotypes, indicating their contributions as primary drivers.

In the forest plot displaying aORs with 95% CIs, high-exposure groups (>P50) are compared against low-exposure groups (<P50, reference) by the median level. Urinary paraben concentrations were log10-transformed and creatinine-corrected. Models are adjusted for age, sex, parental education, parental allergy history, residence, air purifier use, and bedroom cleaning frequency.

**Figure 5:**
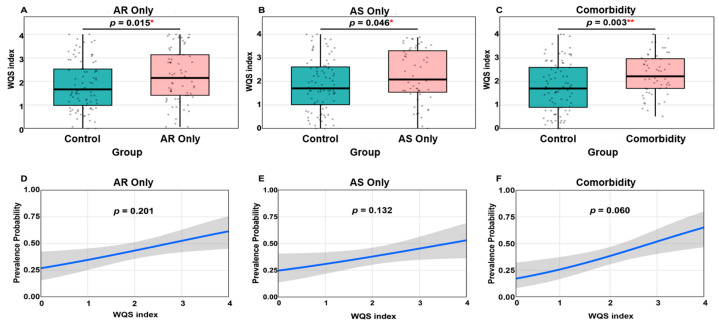
BKMR analysis of urine paraben mixture effects. (**A**–**C**) Boxplots comparing the WQS Index distribution between controls and disease subgroups (AR Only, AS Only, AR&AS Comorbidity). *p*-values are derived from the Wilcoxon Rank-Sum test. (**D**–**F**) Estimated prevalence probability (risk) curves as a function of the WQS Index, with *p*-values indicating the significance of the mixture effect in the regression models. * *p* < 0.05; ** *p* < 0.01.

In the WQS regression, the WQS index was significantly elevated in all phenotype subgroups compared to controls (Figure 5A–C), which revealed the increased cumulative effect of the urine paraben mixture. Using regression models, a positive trend between the WQS index and the risk of each phenotype was observed (Figure 5D–F). The association approached statistical significance for the WQS index and the AR&AS Comorbidity group (*p* = 0.060). Collectively, the above two analyses confirmed that the combined urine paraben exposures, driven largely by PrP and BzP, were associated with increased prevalence of AR Only, AS Only, and AR&AS Comorbidity.

### 3.4. Dust Parabens and Association with Respiratory Allergic Phenotypes

**Figure 6:**
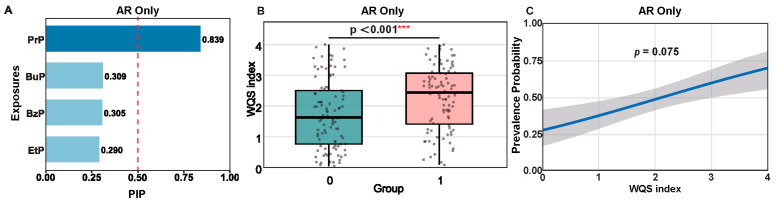
Mixture effects of dust parabens on AR Only. (**A**) BKMR analysis showing Posterior Inclusion Probability (PIP) values, identifying PrP as the primary contributor. (**B**) Distribution of the dust WQS index in Control vs. AR Only groups. (**C**) WQS regression showing a positive association between the dust paraben mixture and the probability of AR (*p* = 0.075). The dashed line indicates a PIP threshold of 0.50. Dark blue bars represent the key contributors (PIP ≥ 0.50) to the mixture effect, while light blue bars indicate variables with lower contribution (PIP < 0.50). *** *p* < 0.001.

We found that, as in urine, PrP showed the highest detection frequency and concentrations in dust. However, dust and urinary concentrations were not significantly correlated, suggesting different exposure sources and routes. Therefore, the impact of dust parabens on children’s respiratory allergies was investigated in parallel. Unlike the strong associations observed in urine parabens, the adjusted significant links between dust parabens and phenotypes appeared only for PrP with “AR Only” (aOR = 2.26, 95% CI: 1.16–4.43, *p* = 0.017). The associations for other compounds and phenotypes were generally weak and nonsignificant in magnitude (Table S5). This finding was corroborated by BKMR analysis, where dust PrP showed a high PIP 0.839 for AR Only (Figure 6A). Additionally, the WQS index for dust parabens was significantly higher in the AR Only group (*p* < 0.001) and positively associated with disease probability (*p* = 0.075).

### 3.5. Interaction Between Paraben Exposure and Parental History of Allergy

Given the significant disparity in the parental history of allergy between case and control groups (Table 1), we further investigated whether this hereditary factor modified the impact of urine paraben exposure. Interaction analysis revealed that this modification effect was particularly prominent in the AR&AS Comorbidity subgroup. In this group, significant interactions were observed on both multiplicative and additive scales (Table S6). For urinary PrP, BzP, and EtP, the positive AP values (0.70, 0.55, and 0.69, respectively) indicated that over half of the estimated risk was attributable to the interaction between paraben exposure and parental allergy history.

Notably, the interaction effect extended to dust paraben exposure. While dust EtP did not show a standalone significant association with AS Only, its interaction with parental allergy history was profound within the AS Only subgroup (AP = 0.64, 95% CI: 0.06–1.22). A similar synergistic pattern was observed for dust BzP and BuP in the AR Only subgroup (AP = 0.56 for both), suggesting that dust-borne parabens may act as potent environmental triggers specifically for children with a hereditary predisposition.

These findings collectively imply a heredity–environment interplay where genetic susceptibility enhances biological vulnerability to paraben-induced allergic outcomes. The magnitude of this synergy appears to be outcome-specific and exposure-route-dependent.

## 4. Discussion

This study quantified four typical parabens on their internal levels in urine samples and external levels in the children’s home dust. Through a case–control study design, urine PrP and BzP were identified as the two dominant compounds associated with children’s multiple respiratory Allergic Phenotypes, including asthma, allergic rhinitis, and comorbidity of both. The BKMR analysis in the mixture of urine parabens corroborated this finding. The WQS index showed that the mixture of four urine parabens exhibited positive associations with phenotypes. The above links were enhanced in subjects with a parental history of allergies, reaching a synergistic effect. Parabens in dust had few significant associations, except for PrP and “AR Only”. Our study exhibited a compound-dependent, heredity-enhanced association between urine paraben exposure and children’s respiratory allergies. Despite the limitation in concluding causal effects, it provided new evidence in understanding the exposure to the new emergent environmental chemicals linked to childhood respiratory allergies.

PrP was detectable in all urine and dust samples in our study, and our samples were collected from a wide range of areas in Shanghai, covering both urban and suburban districts. This universal detection of PrP aligned with comparative analyses of urinary parabens between the United States and China, suggesting that the reliance on propyl-parabens in consumer products remains widespread [39]. The median concentration of PrP in our study was lower than certain historical Chinese aggregate data, but it remained elevated compared to certain European populations [12]. This geographical variation likely reflected divergent regulatory frameworks. While the European Union has implemented stringent restrictions on propyl- and butyl- isoforms in leave-on products for young children (EU Regulation 1223/2009), the continued use of PrP as a cost-effective broad-spectrum antimicrobial in PCPs, particularly in leave-on cosmetics where its moderate lipophilicity facilitates dermal penetration and processed foods in Asian markets, might contribute to the sustained body burden observed in this population [12,40,41].

PrP was significantly associated with almost all respiratory phenotypes and remained robust after adjusting for potential confounders. The strong association indicated that PrP might have a systemic stimulating effect and trigger inflammatory responses in the respiratory tract. Unlike shorter-chain homologs like MeP and EtP, which are lower in endocrine potency, or longer-chain homologs like BuP, which have lower solubility, PrP strikes an optimal balance between antimicrobial efficacy and formulation stability [42]. Mechanistically, PrP disrupts immune homeostasis through estrogenic signaling, promoting Th2-type skewing and elevated IgE levels [1,23,43]. Furthermore, PP-induced oxidative stress impairs airway epithelial integrity, amplifying inflammatory signals and lowering the threshold for allergic airway inflammation [44]. This dual systemic and local disruption drives respiratory pathogenesis.

For BzP, our subjects exhibited a detection frequency exceeding 70% among cases and controls, which was in contrast to earlier studies of Chinese children, where the DRs were typically below 15% [39,45]. This elevation paralleled patterns recently observed in Shanghai adults, suggesting increased exposure might be linked to the high level of industrialization and unique consumption patterns in this metropolis [39]. Notably, in the Chinese urban context, higher SES and purchasing power are associated with increased PCPs consumption and, consequently, higher paraben exposure [46,47]. This elevated purchasing power for preservative-containing PCPs, combined with a fast-paced lifestyle reliant on ultra-processed and plastic-packaged foods, likely shapes this distinctive metropolitan exposure profile [48,49]. In addition, the relatively frequent detection of BzP in indoor dust suggests that household product use and dust-mediated exposure may be important sources of BzP in the Chinese domestic environment [50,51].

More intriguingly, the risk profile for BzP was distinctly targeted in the “AR Only” and “Comorbidity” subgroups. This finding offers environmental epidemiological support for the UAD hypothesis, which posits that the upper and lower respiratory tracts are not anatomically isolated but rather function as a contiguous organ sharing common inflammatory cascades [52,53]. These comorbid patients typically manifest a more severe clinical phenotype than those with a single condition [54,55,56,57,58]. Mechanistically, previous studies have identified epithelial barrier defects in diseases such as asthma and rhinitis, attributing them to genetic factors, exposure to toxic substances that affect the epithelial barrier, and the influence of immune cells and cytokines related to type 2 responses, particularly IL-4 and IL-13 [59,60]. Consequently, children presenting with both AS and AR often suffered from a more profound systemic inflammatory baseline and compromised epithelial barriers [61,62,63]. From a toxicological perspective, BzP is distinct from alkyl esters (like EtP/PrP); its benzyl moiety may confer higher estrogenic potency or stronger receptor binding affinity [14,21,39,64]. We hypothesize that once EDCs like BzP are absorbed by the body, the cumulative inflammatory burden in children with combined respiratory diseases may lower the threshold at which symptoms manifest in nasal and bronchial tissue [26,65]. Notably, due to the cross-sectional nature of the study design, we could not determine the temporal sequence between BzP exposure and the onset of respiratory allergic diseases. The robust ORs observed for BzP in the Comorbidity group, despite the smaller sample size, underscored the importance of distinguishing this phenotype. Pooling these children with pure AS or AR cases could dilute the signal and obscure the true toxicity of specific congeners. The higher prevalence of air purifier use in the comorbid group may also reflect reverse causality, with affected households being more likely to adopt air purifiers in response to existing symptoms rather than representing a causal determinant.

Recognizing that children are exposed to chemical cocktails rather than single agents, the mixture analyses serve to validate and extend our single-pollutant findings. Both BKMR and WQS models consistently identified PrP and BzP as the primary drivers of the mixture effect, lending robustness to the possible exposure–effect relationship. This consistency across different statistical approaches minimized the likelihood that the observed associations were spurious. The positive monotonic relationship between the WQS index and outcome risk, particularly the near-significant trend in the Comorbidity group, supports a “load-dependent” model of toxicity [43,66]. This implied that the developing immune system may be able to buffer low-level exposures, but as the cumulative burden of immunotoxicants like PrP and BzP increases, homeostatic mechanisms may be overwhelmed. This cumulative effect is particularly relevant for pediatric populations, whose immune and respiratory systems are in critical windows of development, rendering them more susceptible to the combined insults of multiple endocrine disruptors [67].

The interaction analysis adds a critical layer of biological complexity to our results, directly linking the environmental findings back to the demographic risk factors. The significant positive interaction between paraben exposure and parental allergy history, particularly on the additive scale (RERI > 0, AP > 0), suggested a biological synergism or a “Double Hit” phenomenon. This finding contextualized the demographic observation that parental history is a strong predictor of disease.

Mechanistically, this interaction is biologically plausible. Children with a family history of atopy often inherit genetic defects in skin barrier function, such as loss-of-function mutations in the filaggrin gene (FLG), which could increase the transdermal absorption of chemicals found in lotions and creams [68,69,70,71]. Furthermore, these children might inherit a dysregulated immune system characterized by a Th2-skewed cytokine profile, making them more reactive to chemical insults [72]. Previous studies have reported associations between paraben exposure and inflammatory biomarkers like IL-4, suggesting that parabens could exacerbate this pre-existing immune dysregulation [24]. Additionally, genetic polymorphisms in carboxylesterase (CES), the major enzyme responsible for hydrolyzing parabens, could result in slower metabolism and higher internal doses in susceptible individuals [73,74]. Notably, our observation that over 50% of the risk for the comorbid (AR&AS) phenotype is attributable to this interaction provides compelling quantitative evidence for this effect. Consequently, this heredity-environment interaction highlighted that safety thresholds derived from the general population may be insufficiently protective for children with atopic heredity, underscoring the critical need to incorporate individual susceptibility into environmental risk assessments and to implement stratified prevention strategies.

Finally, the divergence between urinary and dust biomarkers offered unique insights into exposure pathways and the site of toxicity. Notably, no significant correlation was observed between concentrations in the dust and urine, suggesting that these two matrices might represent different exposure windows or pathways. Regarding associations with health outcomes, while fewer significant associations were detected in dust parabens and disease outcomes, the robust, significant findings in urinary compounds might better capture the biologically active dose relevant to systemic allergic responses. Dust-borne PrP (reflecting external dose) was specifically and exclusively associated with AR Only, in addition to its urinary levels (reflecting systemic dose) in association with AS Only and comorbidities. We propose a “Portal of Entry” effect to explain this specificity. AR is primarily a disease of the nasal mucosa, the direct port of entry for inhaled particles. PrP, having moderate volatility and high usage volume, partitions significantly into indoor dust [75,76]. Compared to lighter parabens that remain strictly in the gas phase and heavier congeners that bind tightly to surfaces, PrP possesses a unique octanol-air partition coefficient (K_oa_) that favors its accumulation in settled dust particles, making dust a specific reservoir for this congener [77]. Inhaled dust particles laden with PrP are likely to deposit on the turbinates and nasal epithelium [78]. Indoor settled dust collected by vacuum sampling predominantly consists of 10–250 µm coarse particles, which are efficiently captured in the nasal passages via inertial impaction at the nasal vestibule and turbinates [79,80]. In practice, larger dust particles can be resuspended and fragmented into smaller airborne particles, particularly within the inhalable coarse range, which are more relevant for nasal deposition. Only particles smaller than approximately 2–3 µm can bypass nasopharyngeal filtration and reach the distal lower airways [81]. Here, they may create a localized zone of high concentration, provoking upper airway inflammation via direct contact, independent of systemic absorption or blood levels [82]. Conversely, AS is a disease of the lower airways. The dust particles large enough to settle (and thus be collected in vacuum samples) are often filtered by the nasopharynx and do not reach the bronchi [83]. However, parabens that are absorbed into the blood (whether through diet, dermal application, or inhalation) can circulate to the pulmonary vasculature, inducing inflammation in the lower respiratory tract [84]. This distinction highlights the necessity of multi-matrix sampling in environmental epidemiology: urine captures the systemic risk driving AS and complex phenotypes, while dust captures the inhalational risk specifically driving rhinitis.

Several limitations warrant consideration when interpreting these results. First, the case–control design precluded definitive causal inference; while we conceptually adjusted for reverse causation and considered the biological plausibility of our findings, longitudinal data are needed to rigorously confirm the temporal sequence of exposure and disease onset. Second, the reliance on single-spot urine samples might not perfectly capture long-term exposure due to the short half-lives of parabens. However, given that PCP usage in children was often habitual and repetitive, spot samples could offer reasonable temporal stability. Third, while we adjusted for major confounders, residual confounding by other co-occurring EDCs (e.g., phthalates, bisphenols) or indoor allergens could not be entirely ruled out. Finally, since children are typically exposed to complex mixtures of environmental chemicals in real-world settings, co-exposures to other EDCs such as phthalates and bisphenols may exist and could potentially influence the observed associations. Future research should aim to incorporate longitudinal birth cohorts with repeated measures and broader chemical screens to further disentangle the complex web of environmental determinants of allergic disease.

## 5. Conclusions

In conclusion, this study suggests that PrP and BzP are associated with pediatric respiratory allergic phenotypes. Our dual-matrix analysis indicated distinct exposure patterns: urinary biomarkers were associated with asthma and comorbidities, whereas dust-borne PrP was linked to allergic rhinitis. Furthermore, interaction analysis suggested that parental allergy history may modify susceptibility to these exposures. These findings highlight the importance of considering specific congeners, genetic background, and multi-matrix sampling in future environmental risk assessments, as combining internal and external exposure data may improve exposure characterization in vulnerable pediatric populations.

## Figures and Tables

**Table 1:** Demographic and household characteristics of the case and control participants in Shanghai. BMI^a^: body mass index.

Characteristics	Control	All Cases		AR Only		AS Only		AR&AS Comorbidity	
	*n* = 120	*n* = 246	*p*	*n* = 105	*p*	*n* = 66	*p*	*n* = 75	*p*
**Age, mean ± SD, years**	6.91 ± 2.64	7.15 ± 2.57	0.415	7.51 ± 2.57	0.089	6.45 ± 2.63	0.256	7.26 ± 2.41	0.358
**Weight, means ± SD, kg**	27.29 ± 11.00	28.03 ± 11.70	0.569	28.75 ± 11.95	0.346	25.86 ± 12.02	0.415	28.91 ± 10.96	0.321
**Height, means ± SD, cm**	123.70 ± 17.34	124.83 ± 17.27	0.558	126.06 ± 18.14	0.322	120.75 ± 16.59	0.269	126.56 ± 16.21	0.253
**BMI^a^, means ± SD, kg/m^2^**	17.35 ± 4.37	17.56 ± 5.25	0.709	17.90 ± 6.74	0.467	17.12 ± 3.98	0.725	17.47 ± 3.65	0.851
**Gender, *n* (%)**			**0.019**		**0.034**		**0.031**		0.376
Male	64 (53.8)	165 (67.1)		72 (68.6)		47 (71.2)		46 (61.3)	
Female	55 (46.2)	81 (32.9)		33 (31.4)		19 (28.8)		29 (38.7)	
**Residency area, *n* (%)**			0.351		0.976		0.380		0.200
Rural	33 (27.5)	81 (32.9)		30 (28.6)		23 (34.8)		28 (37.3)	
Urban	87 (72.5)	165 (67.1)		75 (71.4)		43 (65.2)		47 (62.7)	
**Parents’ Education Level, *n* (%)**			**<0.001**		**0.001**		0.071		**0.009**
Undergraduate and below	66 (55.0)	182 (74.0)		80 (76.2)		46 (69.7)		56 (74.7)	
Above Undergraduate	54 (45.0)	64 (26.0)		25 (23.8)		20 (30.3)		19 (25.3)	
**Parental History of Allergies, *n* (%)**			**<0.001**		**<0.001**		**0.001**		**<0.001**
No	86 (71.7)	98 (39.8)		42 (40.0)		31 (47.0)		25 (33.3)	
Yes	34 (28.3)	148 (60.2)		63 (60.0)		35 (53.0)		50 (66.7)	
**Single Child, *n* (%)**			0.183		0.351		0.247		0.398
Yes	67 (56.8)	159 (64.6)		67 (63.8)		44 (66.7)		48 (64.0)	
No	51 (43.2)	87 (35.4)		38 (36.2)		22 (33.3)		27 (36.0)	
**Breastfeeding duration, *n* (%)**			0.089		0.187		0.445		0.079
0 month	10 (8.3)	39 (15.9)		16 (15.2)		9 (13.6)		14 (18.7)	
<6 months	32 (26.7)	71 (28.9)		31 (29.5)		19 (28.8)		21 (28.0)	
≥6 months	78 (65.0)	136 (55.3)		58 (55.2)		38 (57.6)		40 (53.3)	
**Frequency of using air purifiers, *n* (%)**			0.094		0.103		0.972		0.062
Rarely or Never Used	69 (57.5)	117 (47.6)		48 (45.7)		37 (56.1)		32 (42.7)	
Frequently Used	51 (42.5)	129 (52.4)		57 (54.3)		29 (43.9)		43 (57.3)	
**Frequency of Bedroom Cleaning, *n* (%)**			0.283		0.452		0.056		0.803
Once per day	42 (35.0)	78 (31.7)		32 (30.5)		23 (34.8)		23 (30.7)	
2–3 times per week	45 (37.5)	80 (32.5)		36 (34.3)		15 (22.7)		29 (38.7)	
≤Once per week	33 (27.5)	88 (35.8)		37 (35.2)		28 (42.4)		23 (30.7)	
**Bedroom flooring materials, *n* (%)**			0.560		0.839		0.487		0.223
Wooden Floor	82 (68.3)	159 (64.6)		74 (70.5)		41 (62.1)		44 (58.7)	
Non-Wooden Floor	38 (31.7)	87 (35.4)		31 (29.5)		25 (37.9)		31 (41.3)	
**Bedroom wall Materials, *n* (%)**			0.864		0.754		0.616		0.827
Wallpaper	20 (16.7)	39 (15.9)		22 (21.0)		8 (12.1)		9 (12.0)	
Water-based paint/Emulsion paint, ***n* (%)**	57 (47.5)	111 (45.1)		45 (42.9)		29 (43.9)		37 (49.3)	
Whitewashed wall	31 (25.8)	64 (26.0)		25 (23.8)		19 (28.8)		20 (26.7)	
Others	12 (10.0)	32 (13.0)		13 (12.4)		10 (15.2)		9 (12.0)	
**Furry pet, *n* (%)**			0.429		0.384		0.909		0.579
No	95 (79.2)	184 (74.8)		77 (73.3)		51 (77.3)		56 (74.7)	
Yes	25 (20.8)	62 (25.2)		28 (26.7)		15 (22.7)		19 (25.3)	
**Parental history of smoking, *n* (%)**			**0.041**		0.100		0.271		0.062
No	80 (67.8)	137 (55.9)		59 (56.2)		38 (58.5)		40 (53.3)	
Yes	38 (32.2)	108 (44.1)		46 (43.8)		27 (41.5)		35 (46.7)	

## Data Availability

The data presented in this study are available on request from the corresponding author to protect the privacy of the participants.

## Supplementary Materials

The following supporting information can be downloaded at: https://www.mdpi.com/article/10.3390/toxics14040281/s1. Table S1: The mobile phase gradient used in the separation of target analytes in urine samples. Table S2: Mass spectrometric information of the target analytes. Table S3: Method Validation Parameters for Target Parabens in the Dust and Urine Matrices. Table S4: Concentration Distribution of Target Parabens in All Case and Control Groups. Table S5: Logistic Regression Analysis of the Association between Target Paraben Exposure and Health Outcomes. Table S6: Interaction between the concentrations of four parabens in urine and parental allergy history in the combined disease group. Figure S1: Directed acyclic graph (DAG) of the hypothesized causal relationships between paraben exposure and pediatric allergic phenotypes. Figure S2: Logistic regression results for bedroom dust parabens and respiratory allergic diseases in children.

## Abbreviations

The following abbreviations are used in this manuscript:

LOD	Limits of detection
LOQ	Limits of quantification
DR	Detection rate
AR	Allergic rhinitis
AS	Asthma
AR&AS	Allergic rhinitis & Asthma
PCPs	Personal care products
EDCs	Endocrine-disrupting chemicals
PrP	Propylparaben
BzP	Butylparaben
EtP	Ethylparaben
BuP	Benzylparaben
ISAAC	International Study of Asthma and Allergies in Childhood
UAD	United Airway Disease
BKMR	Bayesian Kernel Machine Regression
WQS	Weighted Quantile Sum
PIP	Posterior Inclusion Probability
