# Supplementary material for: Exposure to Urinary and Dust Parabens: Compound-Specific Risks for Pediatric Respiratory Allergic Phenotypes
Source: Toxics. 2026 Mar 26;14(4):281. doi: 10.3390/toxics14040281 (PMC13120286; doi:10.3390/toxics14040281)
Supplement: Supplementary file 1 [file toxics-14-00281-s001.zip › toxics-4201076-supplementary.pdf]

Supplementary Materials

Exposure to Urinary and Dust Parabens: Compound-Specific Risks for Pediatric Respiratory Allergic Phenotypes

Tables and Figures

Table S1. The mobile phase gradient used in the separation of target analytes in urine samples

Table S2. Mass spectrometric information of the target analytes

Table S3. Method Validation Parameters for Target Parabens in the Dust and Urine Matrices

Table S4. Concentration Distribution of Target Parabens in All Case and Control Groups

Table S5. Logistic Regression Analysis of the Association between Target Paraben Exposure and Health Outcomes

Table S6. Interaction between the concentrations of four parabens in urine and parental allergy history in the combined disease group

Figure S1. Directed acyclic graph (DAG) of the hypothesized causal relationships between paraben exposure and pediatric allergic phenotypes

Figure S2. Logistic regression results for bedroom dust parabens and respiratory allergic diseases in children

Table S1. The mobile phase gradient used in the separation of target analytes in urine samples

**Urine**			**Dust**		
**Time(min)**	**A**	**B**	**Time(min)**	**A**	**B**
0	90%	10%	0	90%	10%
2	90%	10%	2	90%	10%
3.5	60%	40%	3.5	60%	40%
4.5	30%	70%	4.5	30%	70%
7.5	5%	95%	6.5	5%	95%
8.5	5%	95%	7.5	5%	95%
11	90%	10%	8	90%	10%
13	90%	10%	10	90%	10%

Table S2. Mass spectrometric information of the target analytes

Compound	Compound Name	Molecular Formula	CAS Number	Molecular Weight	Parent ion (m/z)	Cone Voltage (V)	Quantification[a] Ion (m/z)	Collision Energy (eV)	Confirmation[b] Ion (m/z)	Collision Energy (eV)	Retention time (min)
**PrP**	Propylparaben	$C_{10}H_{12}O_3$	94-13-3	180.2	179	-30	92.1	-30	136	-20	5.39
**BzP**	Benzylparaben	$C_{14}H_{12}O_3$	94-18-8	228.24	227.1	-25	92.1	-25	136	15	5.64
**EtP**	Ethylparaben	$C_9H_{10}O_3$	120-47-8	166.17	165	-30	93.1	-20	137.1	-15	5.08
**BuP**	Butylparaben	$C_{11}H_{14}O_3$	94-26-8	194.23	193	-30	92.1	-30	136	-20	5.67

Quantification[a]: multiple reaction monitoring (MRM) used for quantification;

Confirmation[b]: MRM used for identification; c: internal Standard.

Table S3. Method Validation Parameters for Target Parabens in the Urine and Dust Matrices

Sample type	Compound	Standard curve	R^2	LOD[a] (ng/ mL)	LOQ[b] (ng/ mL)	Low level (5 ng/ mL, n = 6)			Medium level ((20 ng/ mL for urine, 10 ng/mL for Dust, n = 6)			High level (40 ng/ mL, n = 6)		
						RSD% intra-day[c]	RSD% inter-day[d]	Recovery(%)	RSD% intra-day[c]	RSD% inter-day[d]	Recovery(%)	RSD% intra-day[c]	RSD% inter-day[d]	Recovery(%)
**Urine**	PrP	y = 101.99x + 1.8015	0.9977	0.0316	0.1053	7.68	8.53	85.46	7.12	7.34	89.36	5.98	5.75	108.06
	BzP	y = 77.369x + 1.6313	0.9975	0.0016	0.0054	11.93	12.69	100.88	8.75	8.97	97.13	6.87	6.64	101.79
	EtP	y = 213.6x + 1.9972	0.9982	0.0907	0.3024	4.33	5.22	114.70	2.34	10.93	108.69	6.58	9.00	116.87
	BuP	y = 66.433x + 0.5441	0.9977	0.0011	0.0035	9.69	9.11	105.59	7.46	9.36	104.60	7.17	13.23	114.44
**Dust**	PrP	y=119.72 x+0.0141	0.9997	0.0030	0.0101	1.80	1.98	129.03	1.57	1.53	113.11	2.17	2.14	105.12
	BzP	y=97.94 x+0.4162	0.9934	0.0190	0.0634	3.77	10.73	110.83	12.43	10.44	102.51	4.04%	4.22	108.67
	EtP	y=167.84 x+0.1089	0.9995	0.0396	0.132	2.52	2.82	110.35	2.48	2.41	103.17	1.27	1.44	103.83
	BuP	y=116.53x+ 0.0267	0.9999	0.0025	0.0084	3.41	4.67	110.90	2.54	2.66	118.56	2.81	3.02	117.62

LOD[a] : limit of detection

LOQ[b]: limit of quantitation

RSD% intra-day[c]: relative standard deviation (RSD %) within days;

RSD% inter-day[d]: relative standard deviation (RSD %) between 3 consecutive days.

Table S4. Concentration Distribution of Target Parabens in Cases and Controls

Sample type	Compound	Median (P_{25}, P_{75})	DR(%)	Median(P_{25}, P_{75})	DR(%)	Median (P_{25}, P_{75})	DR (%)	*p*
		**All samples (n = 275)**		**Cases (n = 182)**		**Controls (n= 93)**		
**Urine (μg/g Crea)**	**PrP**	3.599(1.44,9.379)	100.00	4.085(1.626,11.671)	100.00	2.233(1.092,5.217)	100.00	**0.002**
	**BzP**	0.084(0.013,0.39)	76.73	0.129(0.020,0.418)	79.67	0.037(＜ LOD,0.320)	70.97	**0.011**
	**EtP**	0.639(0.114,3.473)	62.55	0.883(0.130,4.547)	66.48	0.399(0.087,1.479)	54.84	**0.039**
	**BuP**	＜ LOD (＜ LOD,0.697)	41.82	＜ LOD (＜ LOD,0.769)	41.76	＜ LOD (＜ LOD,0.604)	41.94	0.968
		**All sample (n = 314)**		**Cases (n = 213)**		**Controls (n = 101)**		
**Dust (μg/g)**	**PrP**	196.322(60.06,584.816)	94.59	233.404 (69.739, 651.190)	97.18	118.327 (43.173, 386.145)	89.11	**0.003**
	**BzP**	0.273(0.134,13.247)	55.41	7.485 (＜ LOD, 14.800)	53.99	7.171 (＜ LOD, 11.607)	58.42	0.576
	**EtP**	56.149(20.681,184.315)	91.40	67.200 (23.200, 226.048)	90.61	52.584 (20.000, 146.849)	93.07	0.097
	**BuP**	30.677(4.45,36.936)	83.76	30.924 (9.064, 38.100)	85.45	29.058 (1.992, 34.861)	80.19	**0.024**

LOD: Limit of Detection**; DR**: detection rate. The concentration of parabens in the urine samples are expressed as "**μg/g Crea**" which was calculated by dividing raw levels (ppb) by creatinine concentrations (μmol/L), wit unit conversion based on creatinine's molecular mass. **Corrected Concentration (μg/g Crea) = Paraben (ppb) / (Creatinine (μmol/L) *113.12 *10^{-6})**

Table S5. Logistic Regression Analysis of the Association between Paraben Levels and Respiratory Allergic Phenotypes

Compounds	Urine samples				Dust samples			
**All Cases**	**Crude(N=276)**	***p***	**Adjusted(N=270)**	***p***	**Crude(N=314)**	***p***	**Adjusted(N=308)**	***p***
PrP	**2.13(1.28,3.56)**	**0.004**	**2.37(1.32,4.25)**	**0.004**	**1.86(1.15,3.01)**	**0.012**	1.66(0.97,2.84)	0.063
BzP	**2.13(1.28,3.56)**	**0.004**	**2.61(1.33,5.13)**	**0.005**	1.03(0.64,1.65)	0.904	0.89(0.52,1.53)	0.678
EtP	1.53(0.92,2.53)	0.099	1.29(0.73,2.28)	0.383	1.38(0.86,2.22)	0.185	1.19(0.69,2.06)	0.534
BuP	0.97(0.59,1.59)	0.899	1.38(0.78,2.45)	0.267	1.55(0.96,2.5)	0.071	1.44(0.84,2.46)	0.18
**AR Only**	**Crude(N=162)**	***p***	**Adjusted(N=158)**	***p***	**Crude(N=314)**	***p***	**Adjusted(N=308)**	***p***
PrP	**1.92(1.02,3.61)**	**0.044**	**2.18(1.02,4.65)**	**0.043**	**2.27(1.27,4.04)**	**0.005**	**2.26(1.16,4.43)**	**0.017**
BzP	1.81(0.96,3.4)	0.066	**2.53(1.02,6.28)**	**0.046**	1.07(0.61,1.87)	0.826	0.93(0.48,1.8)	0.834
EtP	**1.94(1.03,3.65)**	**0.040**	1.79(0.84,3.8)	0.130	1.36(0.77,2.39)	0.290	1.17(0.59,2.3)	0.657
BuP	0.90(0.48,1.67)	0.733	1.45(0.68,3.07)	0.335	1.75(0.99,3.1)	0.054	1.51(0.78,2.93)	0.221
**AS Only**	**Crude(N=148)**	***p***	**Adjusted(N=144)**	***p***	**Crude(N=314)**	***p***	**Adjusted(N=308)**	***p***
PrP	**2.31(1.17,4.55)**	**0.016**	**2.92(1.33,6.42)**	**0.008**	1.52(0.79,2.95)	0.209	1.7(0.8,3.6)	0.17
BzP	**2.14(1.09,4.22)**	**0.028**	2.30(0.92,5.79)	0.077	0.95(0.49,1.83)	0.877	0.88(0.42,1.85)	0.737
EtP	1.1(0.56,2.16)	0.772	1.14(0.54,2.41)	0.735	1.34(0.69,2.57)	0.385	1.36(0.66,2.81)	0.41
BuP	1.17(0.6,2.29)	0.637	1.58(0.74,3.39)	0.24	1.09(0.56,2.1)	0.802	1.39(0.67,2.88)	0.381
**Comorbidity**	**Crude(N=152)**	***p***	**Adjusted(N=146)**	***p***	**Crude(N=314)**	***p***	**Adjusted(N=308)**	***p***
PrP	**2.25(1.16,4.38)**	**0.017**	1.93(0.86,4.34)	0.113	1.67(0.89,3.14)	0.109	1.48(0.71,3.09)	0.298
BzP	**2.59(1.33,5.07)**	**0.005**	**3.55(1.32,9.55)**	**0.012**	1.05(0.56,1.96)	0.874	1.07(0.51,2.25)	0.861
EtP	1.57(0.81,3.03)	0.178	1.09(0.48,2.48)	0.837	1.45(0.78,2.71)	0.243	1.08(0.5,2.33)	0.845
BuP	0.88(0.46,1.7)	0.711	1.27(0.58,2.82)	0.549	1.78(0.95,3.35)	0.072	1.55(0.74,3.26)	0.249

In the adjusted model,age, gender, residence (urban/rural), parental education, parental allergy history, air purifier usage frequency, and bedroom cleaning frequency.

Table S6. Interaction between the concentrations of four parabens in urine and dust and parental allergy history in the three disease groups.

Indicator	PrP	BzP	EtP	BuP
	**Urine**			
**AR Only**				
Multiplicative scale	1.24 [0.28, 5.51]	0.82 [0.18, 3.75]	1.79 [0.4, 8.14]	2.35 [0.49, 11.22]
RERI[a]	2.81 [-4.21, 9.83]	5.95 [-7.76, 19.67]	4.28 [-3.21, 11.77]	4.49 [-3.74, 12.72]
AP[b]	0.39 [-0.3, 1.08]	0.45 [-0.15, 1.05]	**0.55 [0.02, 1.07]**	**0.58 [0.06, 1.1]**
SI[c]	1.82 [0.46, 7.28]	1.95 [0.58, 6.61]	2.67 [0.59, 12.11]	3.04 [0.58, 15.93]
**AS Only**				
Multiplicative scale	0.85 [0.18, 4]	1.06 [0.23, 4.82]	1.05 [0.23, 4.81]	1.06 [0.2, 5.44]
RERI[a]	2.43 [-5.11, 9.97]	2.55 [-4.48, 9.58]	0.02 [-3.94, 3.98]	1.49 [-4.98, 7.96]
AP[b]	0.32 [-0.43, 1.08]	0.37 [-0.34, 1.09]	0.01 [-1.24, 1.26]	0.27 [-0.66, 1.2]
SI[c]	1.59 [0.42, 6.06]	1.78 [0.43, 7.3]	1.01 [0.16, 6.41]	1.49 [0.3, 7.5]
**Comorbidity**				
Multiplicative scale	2.49 [0.5, 12.44]	0.81 [0.16, 4.17]	**6.14 [1.16, 32.46]**	1.78 [0.34, 9.2]
RERI[a]	10.38 [-4.6, 25.36]	13.61 [-13.98, 41.2]	4.88 [-1.92, 11.68]	3.78 [-5.93, 13.48]
AP[b]	**0.7 [0.35, 1.05]**	**0.55 [0.05, 1.05]**	**0.69 [0.24, 1.13]**	0.41 [-0.29, 1.12]
SI[c]	3.96 [0.97, 16.12]	2.34 [0.72, 7.57]	4.94 [0.42, 57.55]	1.87 [0.45, 7.78]
	**Dust**			
**AR Only**				
Multiplicative scale	0.81 [0.21, 3.13]	3.5 [0.9, 13.54]	0.98 [0.25, 3.77]	2.42 [0.64, 9.15]
RERI[a]	4.37 [-4.55, 13.29]	2.82 [-1.46, 7.1]	0.59 [-4.82, 6]	3.71 [-1.62, 9.04]
AP[b]	0.4 [-0.16, 0.95]	**0.56 [0.04, 1.07]**	0.1 [-0.8, 1.01]	**0.56 [0.09, 1.03]**
SI[c]	1.77 [0.62, 5.07]	3.28 [0.45, 23.93]	1.14 [0.33, 3.96]	2.93 [0.63, 13.63]
**AS Only**				

Multiplicative scale	0.41 [0.09, 1.84]	1.06 [0.23, 4.82]	3.69 [0.82, 16.65]	0.49 [0.11, 2.14]
RERI[a]	-1.44 [-7.04, 4.16]	2.55 [-4.48, 9.58]	1.9 [-0.99, 4.78]	-1.25 [-6.19, 3.7]
AP[b]	-0.33 [-1.71, 1.05]	0.37 [-0.34, 1.09]	**0.64 [0.06, 1.22]**	-0.33 [-1.77, 1.11]
SI[c]	0.7 [0.19, 2.56]	1.78 [0.43, 7.3]	-	0.69 [0.17, 2.83]
**Comobidity**				
Multiplicative scale	1 [0.23, 4.41]	0.44 [0.1, 1.92]	2.57 [0.58, 11.52]	1.07 [0.25, 4.7]
RERI[a]	0.18 [-6.64, 6.99]	-3.03 [-12.86, 6.8]	4.44 [-2.37, 11.25]	0.64 [-6.15, 7.42]
AP[b]	0.03 [-1.03, 1.08]	-0.41 [-1.85, 1.02]	**0.56 [0.06, 1.05]**	0.1 [-0.87, 1.06]
SI[c]	1.03 [0.29, 3.75]	0.68 [0.22, 2.11]	2.76 [0.61, 12.42]	1.13 [0.32, 3.99]

RERI[a]: Relative Excess Risk due to Interaction; **AP[b]**: Attributable Proportion; **SI[c]**: Synergy Index.

SI for the Dust-AS Only subgroup was omitted due to numerical instability (SI=32.77, 95% CI exceeded 10^{14}) arising from the small denominator in the additive model; interaction interpretation is primarily based on the AP.

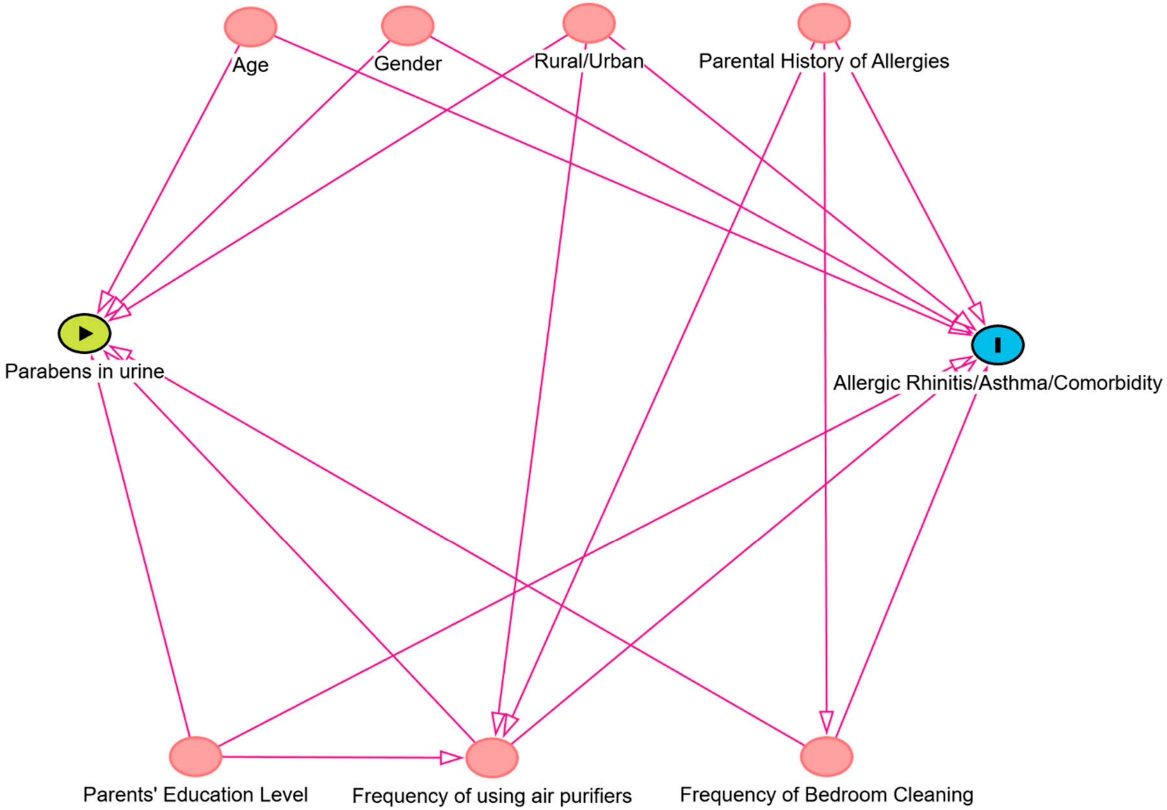

Figure S1. Directed acyclic graph (DAG) of the hypothesized causal relationships between paraben exposure and pediatric allergic phenotypes.

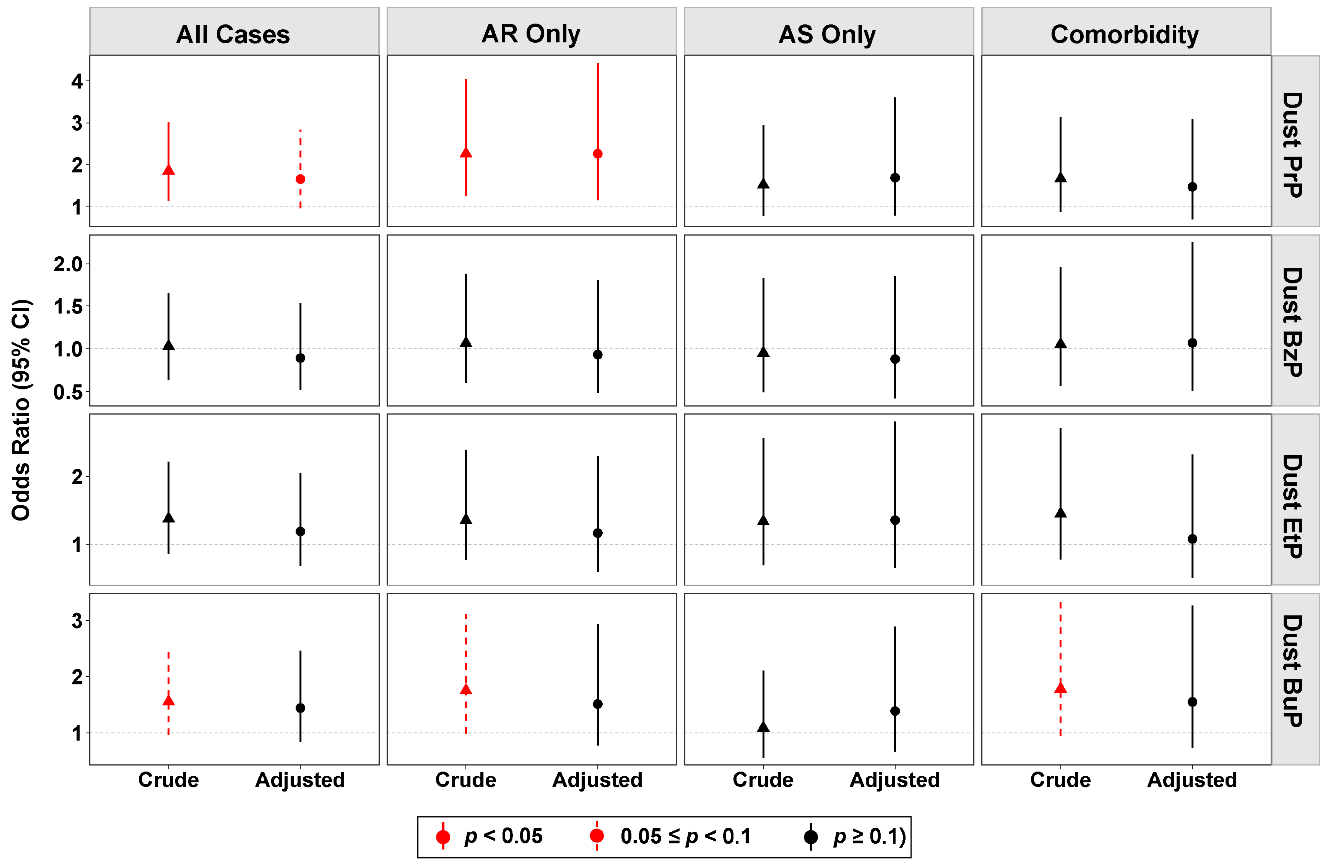

Figure S2. Logistic regression results for bedroom dust parabens and respiratory allergic diseases in children.

Note. The high-exposure group (>P50) was compared against the low-exposure group (< P50) used as the reference. Bedroom dust paraben concentrations were Log_{10}-transsition.
